# Supplementary material for: Single versus Multiple Dose Ivermectin Regimen in Onchocerciasis-Infected Persons with Epilepsy Treated with Phenobarbital: A Randomized Clinical Trial in the Democratic Republic of Congo
Source: Pathogens. 2020 Mar 10;9(3):205. doi: 10.3390/pathogens9030205 (PMC7157533; doi:10.3390/pathogens9030205)
Supplement: Supplementary file 1 [file pathogens-09-00205-s001.zip › S4 Seizure types.docx]

**S4 Table : Seizure characteristics of participants at baseline and during the last four months of the study**

|  | Enrolled at baseline  **N = 197** | Completed last four months’ evaluation  **N = 157** | Premature discontinuation of the study*  **N = 40** | | |
| --- | --- | --- | --- | --- | --- |
| ***Seizure Types*** | Baseline Seizures:  n (% participants) | Seizure-free during the last four months:  n (% baseline) | Deaths | Lost to follow-up | Withdrew consent |
| Tonic-clonic only | 105 (53.3%) | 48 (45.7%) | 6 | 9 | 5 |
| Atonic only | 1 (0.5%) | 0 (0%) | 1 | 0 | 0 |
| Absence seizures only | 4 (2%) | 2 (50%) | 0 | 2 | 0 |
| Nodding seizures only | 1 (0.5%) | 0 (0%) | 0 | 0 | 0 |
| Absences+Tonic-clonic seizures | 70 (35.5%) | 31 (44.3) | 3 | 5 | 4 |
| Nodding+Tonic-clonic seizures | 4 (2%) | 1 (25%) | 0 | 1 | 0 |
| Nodding+Absence seizures | 4 (2%) | 1 (25%) | 1 | 0 | 0 |
| Other seizures types (Focal, etc) | 8 (4.1%) | 2 (25%) | 1 | 2 | 0 |
| ***Total*** | 197 | 85 | 12 | 19 | 9 |
| **Considered as not having achieved seizure freedom (treatment failure)* | | | | | |
