# Supplementary material for: Single versus Multiple Dose Ivermectin Regimen in Onchocerciasis-Infected Persons with Epilepsy Treated with Phenobarbital: A Randomized Clinical Trial in the Democratic Republic of Congo
Source: Pathogens. 2020 Mar 10;9(3):205. doi: 10.3390/pathogens9030205 (PMC7157533; doi:10.3390/pathogens9030205)
Supplement: Supplementary file 1 [file pathogens-09-00205-s001.zip › S2 Clinical Trial_Protocol.docx]

**Supplementary material**

**S2: Clinical trial protocol**

**Single versus multiple dose ivermectin regimen in onchocerciasis-infected persons with epilepsy treated with phenobarbital: a randomized clinical trial in the Democratic Republic of Congo**

**Amendement au protocole «Traitement à l’Ivermectine de patients avec Epilepsie associée à l’Onchocercose: Un essai clinique randomisé et contrôlé (EAOTRAIT)»**

**EAOTRAIT extra Protocol version 1.1**

4.09.2017

| **Nom et Contacts de l’Investigateur Principal DRC** | Michel MANDRO NDAHURA, Division Provinciale de la Santé de l’Ituri, RDC  Email : [mandhura@yahoo.fr](file:///G:\A%20faire\mandhura@yahoo.fr)  Téléphone : (243) 97 65 00 675  (243) 82 40 65 190 |
| --- | --- |
| **Investigateur Principal Belge** | Robert Colebunders, Global Health Institute, University of Antwerp, Antwerp, Belgium  Email : [bcoleb@itg.be](file:///G:\A%20faire\bcoleb@itg.be)  Téléphone : (32) 486920149 |
| **Site de recherche  et localisation** | Centre de Recherche en Maladie Tropicale de l’Ituri (CRMT)  Hôpital General de Référence de Rethy  Zone de Sante de Rethy  Province de l’Ituri  République Démocratique du Congo |
| **Adresse postale de l’institution** | Division Provinciale de la Santé de l’Ituri  Bunia, Quartier Mudzi Pela, Avenue Plateau Médical,  Sous quartier Bigo III  BP: 19, Bunia  Province de l’Ituri |
| **Co-Investigateurs principaux** | Naomie Awaca. Directrice du Programme National de lutte contre les Maladies Tropicales Négligées-CTP/RDC ; Ministère de la santé  Deby Mukendi, Centre Neuro Psycho Pathologique, Université de Kinshasa, République Démocratique du Congo  Dolo Husseini Global Health Institute, University of Antwerp, Antwerp, Belgium  Patrick Suykerbuyk Global Health Institute, University of Antwerp, Antwerp, Belgium |

| **Investigateurs Principaux** | **Signature** |
| --- | --- |
| **Michel Mandro** |  |
| **Robert Colebunders** |  |

**Introduction**

Des études récentes au Cameroun ont démontré que le traitement par ivermectine réduit l’incidence de l’épilepsie lié à l’onchocercose (R Colebunders, publication en préparation) . Par ailleurs une étude par T Johnson a identifié des anticorps anti-Leiomodine-1 chez des patients avec hochement de tête qui sont neurotoxique et qui réagissent avec certaines protéines du parasite *Onchocerca volvulus* (1). L’épilepsie lié à l’Onchocercose peut donc être une maladie auto-immune initiée par l’*Ochocerca volvulus*. Des maladies auto-immunes sont en général des maladies avec une évolution progressive. Dans le cadre de l’épilepsie liée à l’onchocercose on pourra espérer que la maladie ne sera pas progressive si on peut éliminer le facteur qui déclenche l’auto-immunité: l’*Onchocerca volvulus*. Raison donc d’évaluer l’effet d’un traitement d’ivermectine 3 fois par ans en comparaison avec le traitement du programme de lutte contre l’onchocercose: une fois par ans. En effet avec un traitement de seulement une fois par ans des microfilaires peuvent réapparaitre après 4 mois et donc potentiellement risque de crises d’épilepsie.

**Objectif supplémentaire**

Evaluer chez des personnes avec un épilepsie liée à l’onchocercose l’effet d’un traitement ivermectine 3 fois par ans avec un traitement une fois par ans sur la fréquence des crises d’épilepsie.

**Méthode**

**Site de l’étude**

Villages de la Zone de santé de Logo, dans la province de l’Ituri, République Démocratique du Congo.

## **Définitions**

### Définition de l’infection à *Onchocerca volvulus*

Une biopsie cutanée positive pour microfilaires à *Onchocerca volvulus* et/ou une sérologie positive au test anticorps Ov16.

### Diagnostic de l’épilepsie

Un cas d’épilepsie sera défini comme au moins 2 épisodes de crises d’épilepsie non-provoquées, non-associées de fièvre.

**Critères d’Inclusion :**

1. Les personnes déjà incluses dans le protocole initial (groupe 1)

2. Les personnes supplémentaires (groupe 2):

**Critères d’évaluation de l’étude**

La proportion de patients qui ne présenteront aucune crise d’épilepsie dans les 4 derniers mois de suivi.

**Randomisation et schéma de suivi**

1. Groupe 1A (groupe «ivermectine immédiat») et 1B (groupe «ivermectine retardé») de l’étude de 4 mois seront répartis en 4 groupes : 1Aa, 1Ab, 1Ba et 1Bb, mais qui seront randomisés 4 mois après l’inclusion dans l’étude EAOTRAIT dans 2 nouveau bras de traitement suivants : a) ivermectine une fois + continuer traitement anti-épileptique pour 1Ab et 1Bb et b) ivermectine chaque 4 mois + continuer traitement anti-épileptique pour 1Aa et 1Ba. ( voir le schéma ci bas).

2. Groupe 2 sera randomisé dans un bras a) ivermectine deux fois + traitement anti-épileptique et un bras b) ivermectine chaque 4 mois + traitement anti-épileptique ( voir le schéma ci bas).

**(Mois)**

**(Semaines)**

**Groupe 2A**

**Groupe 2B**

Départ

J1/S1

**IV1**

et TAE

**IV1**

et TAE

M1

S1-S4

TAE

TAE

M2

S5-S8

TAE

TAE

M3

S9-S12

TAE

TAE

M4

S13-S16

TAE

TAE

**GROUPE 1Aa**

**GROUPE 1Ab**

**GROUPE 1Ba**

**GROUPE 1Bb**

J1/S17

**IV2**

et TAE

TAE

**IV1**

et TAE

**IV1**

et TAE

**IV2**

et TAE

**IV2** et TAE

M5

S17-S20

TAE

TAE

TAE

TAE

TAE

TAE

M6

S21-S24

TAE

TAE

TAE

TAE

TAE

TAE

M7

S25-S28

TAE

TAE

TAE

TAE

TAE

TAE

M8

S29-S32

TAE

TAE

TAE

TAE

TAE

TAE

J1/S33

**IV3**

et TAE

TAE

**IV2**

et TAE

TAE

**IV3**

et TAE

TAE

M9

S33-36

TAE

TAE

TAE

TAE

TAE

TAE

M10

S37-S40

TAE

TAE

TAE

TAE

TAE

TAE

M11

S41-S44

TAE

TAE

TAE

TAE

TAE

TAE

M12

S45-S48

TAE

TAE

TAE

TAE

TAE

TAE

J1/S49

**IV4**

et TAE

TAE

**IV3**

et TAE

TAE

**IV4**

et TAE

TAE

M13

S49-S53

TAE

TAE

M14

S54-56

TAE

TAE

M15

S57-S60

TAE

TAE

M16

S61-64

TAE

TAE

J1/65

**IV4**

et TAE

TAE

**EVALUATION**

**RANDOMIZATION 1'**

TAE

**VALIDES**

I5: épilepsie avec ? 2 crises par mois

I5: épilepsie avec 1 crise

par mois

**GROUPE 1**

**GROUPE 2**

**Groupe 1B**

**IV1**

et TAE

NON-VALIDES

(personne exclue)

**EVALUATION**

**Critères d'inclusion**

**(I1-I4)**

**et exclusion**

**(E1-**

**E8)**

**valide (voir feuille**

**"Critères_INCLUSION_EXCLUSION")?**

**PARTICIPANT POTENTIEL**

**EVALUATION**

**PERIODE**

TAE

TAE

**EVALUATION**

**RANDOMIZATION 2**

**RANDOMIZATION 2'**

TAE

TAE

TAE

TAE

TAE

TAE

**RANDOMIZATION 1**

**Groupe 1A**

IV : Ivermectine (Mectizan) ; TAE : traitement anti-épileptique ; M : mois ; S : semaine ; j : jour

**Taille de l’échantillon**

Si on estime que 60 % des participants du groupe 1 reçoivent de l’ivermectine trois fois par année et que 30 % reçoivent l’ivermectine une fois par ans ne présenteront aucune crise d’épilepsie au cours des quatre derniers mois de l’étude (9 à 12 mois après avoir reçu la première dose d’ivermectine), ce qui implique un rapport de cotes (odd ratio-OR) de 3,5, 84 (2 x 42) participants seront nécessaires pour détecter une différence significative dans les cotes entre les régimes de traitement avec une puissance de 80% et un niveau de signification sur les deux côtés de 5%. Si on estime que 65% des participants du groupe 2 (la plupart d’entre eux ayant moins de crises que ceux du groupe 1) reçoivent l’ivermectine trois fois par an ne présenteront aucune crise au cours des quatre derniers mois de l’étude, par rapport à 40 % de ceux qui reçoivent de l’ivermectine deux fois par ans (OR = 2,5), 123 (2 x 62) participants pourront être nécessaires pour détecter une différence significative avec une puissance de 80 % et un niveau de signification sur les deux côtés de 5 %. Si on estime que le taux de perte lors de suivi ou de retrait prématuré de l’étude soit de 10 %, il faudrait un minimum de 229 participants (soit 94 dans le groupe 1 et 135 dans le groupe 2).

**Calendrier de l’étude group 1**

**
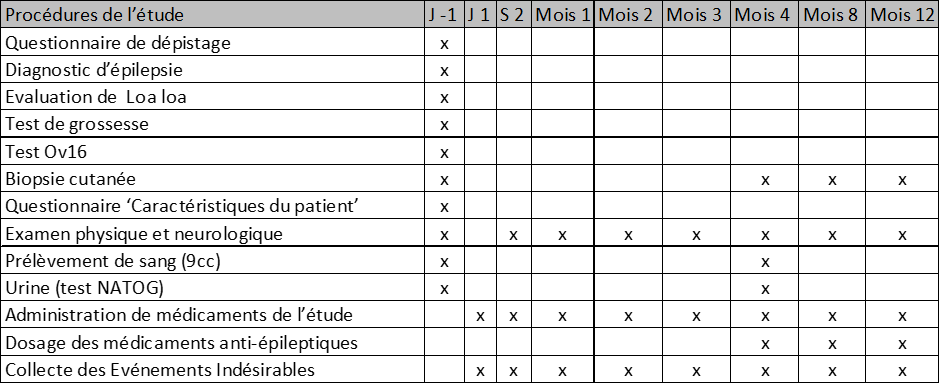
**

**Calendrier de l’étude group 2**

**Gestion des données et plan d’analyse**

Toutes les analyses seront faites sur le principe «intention de traiter»: tous les patients seront inclus dans l’analyse en fonction du bras dans lequel ils ont été randomisés.

La proportion de personnes sans crise d’épilepsie sera comparée dans le bras 1A,Aa + Ba + 2A avec la proportion des réponses observées dans les bras Ab + Bb +2B. Les fréquences de crises seront comparées entre les participants avec et sans biopsie cutanée positive aux microfilaires à 4 et à 8 mois.

Les participants perdus pendant le suivi seront considérés comme non-répondants.

De la même façon, les patients chez qui le régime anti-épileptique a été adapté à cause d’une augmentation du nombre de crises seront considérés comme non-répondants. Les changements de traitement anti-épileptique à cause d’effets indésirables ou la possibilité d’interactions médicamenteuses ne seront pas considérés comme un échec de traitement.

## **Sécurité de l’étude**

L’Ivermectine s’est avérée d’être un médicament idéal pour la distribution de masse pour l’élimination de l’onchocercose. Le médicament a peu d’effets indésirables chez les patients sans co-infection avec *Loa Loa* (non endémique dans la région où l’étude sera conduite).

Après avoir pris l’ivermectine, les personnes qui ont beaucoup de microfilaires dans leur peau peuvent avoir pendant quelques jours des démangeaisons, des éruptions, des douleurs musculaires et articulaires, des douleurs musculaires au niveau abdominal, de la fièvre, un gonflement du visage, des bras ,des mains, des jambes ou des nodules lymphatiques, de la nausée, des vomissements, de la diarrhée, des vertiges, une sensation de faiblesse, de la tachycardie, des céphalées, ou des difficultés de respirer.

Quelques personnes qui ont des microfilaires dans leurs yeux peuvent avoir des sensations anormales dans les yeux, des gonflements des paupières, ou une irritation des yeux qui peut provoquer des difficultés de vision.

Ces manifestations arrivent principalement quand le médicament est utilisé pour la première fois. Il s’agit de réactions allergiques passagères au moment où les microfilaires sont tuées (réaction de Mazotti).En Ouganda la distribution de masse d’ivermectine est faite deux fois par an sans aucun problème. Des interactions entre l’ivermectine et les antiépileptiques n’ont pas été signalées. Les effets secondaires avec ivermectine sont surtout observé pendant la première prise (2). Un traitement d’ivermectine même 4 fois par ans ne pose pas de problèmes d’effets secondaires important sans co-infection au loa Loa (2).

**Considérations éthiques**

L’étude sera conduite en accord avec les lois et règlements applicables incluant, mais non limité , les recommandations de la Conférence Internationale sur l’Harmonisation des Recommandations pour les Bonnes Pratiques Cliniques (BPC) ainsi que les principes cliniques qui ont leurs origines dans la Déclaration d’Helsinki. Le but et la nature des investigations seront expliqués aux participants et aux responsables légaux, y compris les risques et les avantages de chacune des procédures. Si un sujet souhaite participer à l’étude, lui ou elle et (si applicable) le responsable légal devra participer aux procédures de consentement éclairé et un formulaire de consentement éclairé devra être daté et signé, éventuellement par empreinte digitale, par le sujet (et le responsable légal, si applicable) ou par son représentant légal, un témoin alphabétisé (pour les participants illettrés) et par l’investigateur principal ou son délégué, avant que toute procédure liée au protocole de l’étude soit initiée. Chaque participant recevra une fiche d’information détaillée comprenant, notamment mais non limité, à une description de la pathologie, les avantages et les risques potentiels de l’essai, les règles de confidentialité et le devenir des données et des prélèvements. En cas de refus de participation à l’étude, un traitement à l’ivermectine (en cas d’onchocercose), un traitement de l’épilepsie ainsi que l’accès aux médecins de l’étude seront également offerts. Nous collaborer avec l’ organisation humanitaire Maltezer et les services de santé locaux afin d’ assurer qu’après l’étude les participants pourront continuer le traitement antiépileptique et la prise au moins annuelle d’ivermectine. Si l’étude démontre une meilleure activité d’ivermectine 3 fois par ans, on demandera au programme national de lutte contre l’onchocercose de distribuer ce traitement pour les personnes avec une épilepsie associé à l’onchocercose. L’approbation du comité éthique pour cet amendement sera obtenue auprès du comité d’éthique de l’école de Santé Publique de l’Université de Kinshasa et du comité d’éthique de l’Université d’Anvers.

**Financement**

L’étude sera financée par le fond **ERC, No.671055** de Robert Colebunders

**Références**

1. Johnson TP, Tyagi R, Lee PR, Lee MH, Johnson KR, Kowalak J, Elkahloun A, Medynets M, Hategan A, Kubofcik J, Sejvar J, Ratto J, Bunga S, Makumbi I, Aceng JR, Nutman TB, Dowell SF, Nath A. [Nodding syndrome may be an autoimmune reaction to the parasitic worm *Onchocerca volvulus*.](https://www.ncbi.nlm.nih.gov/pubmed/28202777) Sci Transl Med. 2017 Feb 15;9(377).

2. Kamgno J, Gardon J, Gardon-Wendel N, Demanga-Ngangue, Duke BO, Boussinesq M.

[Adverse systemic reactions to treatment of onchocerciasis with ivermectin at normal and high doses given annually or three-monthly.](https://www.ncbi.nlm.nih.gov/pubmed/15186939) Trans R Soc Trop Med Hyg. 2004 Aug;98(8):496-504

**Traitement à l’Ivermectine de patients avec Epilepsie associée à l’Onchocercose: Un essai clinique randomisé et contrôlé (EAOTRAIT extra)**

**NOTE D’INFORMATION DESTINÉE AUX PARTICIPANTS**

Cher Monsieur, chère Madame,

Nous souhaitons vous inviter à participer à une étude sur l’effet de l’ivermectine sur l’épilepsie liée à l’onchocercose (cécité des rivières). Cette ’étude est sponsorisée par le Ministère de la Santé Publique de la RDC et l’Université d’Anvers. La raison qui fait que vous êtes invité à participer à cette étude est que vous souffrez d’épilepsie et que vous vivez dans un village où il existe beaucoup d’onchocercose. Avec cette étude nous voulons évaluer si la prise d’ivermectine pourra diminuer la fréquence des crises d’épilepsie.

Avant de décider si vous (ou le membre malade de votre famille) acceptez de participer ou non à cette étude, il est important que vous compreniez bien les informations dans ce formulaire car il spécifie vos droits et vos responsabilités. Nous vous invitons avant que vous décidiez de participer à cette étude, de prendre tout votre temps pour poser des questions ou discuter avec votre famille, vos amis, et ou les prestataires de santé

Ce document d’information et le Consentement éclairé qui suit vous expliquent également le but de l’étude, les examens nécessaires pour cette étude ainsi que les avantages et les risques liés à votre participation. Votre participation est entièrement volontaire et vous pouvez prendre le temps de réfléchir à votre décision. Cela n’aura pas d’influence sur les soins que vous allez recevoir ici. Ils seront les mêmes, même si vous décidez de ne pas participer. De plus, si vous acceptez, vous pouvez toujours vous retirer de l’étude à tout moment si vous le souhaitez. Le médecin responsable de l’étude est là pour répondre à vos questions, n’hésitez pas à lui en poser.

**Introduction: onchocercose et épilepsie**

Les vers de l’onchocercose peuvent provoquer des démangeaisons cutanées et des lésions oculaires variées. Après de multiples années d'exposition, ces lésions conduisent à une cécité irréversible et à des lésions cutanées inesthétiques appelées parfois "peau de léopard" ou de " lézard". Il est très probable que l’onchocercose peut également causé de l’épilepsie. Les crises d’épilepsie sont provoquées par une décharge électrique excessive soudaine dans certaines zones cérébrales.

L’onchocercose et l’épilepsie existent dans beaucoup de parties du monde y compris la RDC. Le traitement avec l’ivermectine peut réduire voire tuer les vers de l’onchocercose et prévenir la transmission de la maladie à d’autres personnes. Pour cette raison, les autorités sanitaires de la RDC avec l’Organisation Mondiale de la Santé et d’autres partenaires ont commencé la distribution annuelle de masse du traitement avec l’ivermectine dans les villages où l’onchocercose existe. Il se pourrait que ce traitement soit présent dans votre village.

Si une personne souffre d’une épilepsie liée à l’onchocercose, il est possible que l’élimination des vers de l’onchocercose puisse réduire les crises d’épilepsie.

**But et description de l’étude**

Le but de l’étude est de détecter si le traitement avec l’ivermectine pourrait réduire les crises liées à l’épilepsie due à l’onchocercose.

**Conduite de l’étude**

Si vous acceptez de participer à cette étude, le médecin ou l’infirmier ou le personnel scientifique de l’étude commencera par vous interroger et vous examiner. Ensuite, on prélèvera du sang par une piqûre dans le bras et on prélèvera deux petits morceaux de peau au niveau des hanches pour rechercher la présence de vers de l’onchocercose dans la peau.

Vous pouvez participer à l’étude dans les conditions suivantes :

1) Si une infection par l’onchocercose est documentée par un test de sang et/examen de la peau. 2) Si vous souffrez épilepsie active diagnostiquée et confirmée par le médecin de l’équipe.

3) Si vous acceptez d’être suivi à la consultation pendant une période de 12 mois et de subir les tests proposés par le protocole de l’étude.

4) Si vous n’êtes pas enceintes ou n’êtes pas en train d’allaiter votre enfant.

5) Si vous n’avez pas été traité avec l’ivermectine les 9 mois avant le début cette étude.

6) Si vous acceptez que des échantillons de sang, et les échantillons de peau seront envoyés et conservés à Kinshasa et dans des laboratoires étrangers pour permettre des analyses qui ne sont pas possibles ici. Si vous ne souhaitez pas qu’on conserve ces prélèvements, il suffit de le dire au médecin et de ne pas cocher la case dans le formulaire de Consentement.

Si l’épilepsie est confirmée par le médecin, des conseils concernant la prise en charge de l’épilepsie seront donnés et un traitement pour l’épilepsie sera offert. Votre participation à l’étude n’entrainera aucun frais pour vous.

En tout ,nous estimons que de 229 personnes avec une épilepsie et une infection par l’onchocercose seront sélectionnées pour participer dans cette étude. La moitié des personnes choisie au hasard recevra de l’ivermectine directement une fois par ans et l’autre moitié recevra l’ivermectine 3 fois par ans. Au début et après 4 mois et 8 mois, le nombre de vers de l’onchocercose dans la peau sera évalué en faisant un prélèvement de 2 petits morceaux de peau. Egalement au début de l’étude et à 4 et 8 mois, 2 tubes (2 cuillerées à café) de votre sang seront prélevés d’une veine de votre bras et un échantillon d’urine sera demandé pour rechercher des produits excreté par le vers qui cause l’onchocercose. Dans le sang on fera également un dosage de la quantité de médicaments anti-épileptiques.

Tous les participants auront un interrogatoire médical et un examen physique bref au début de l’étude.

Chaque participant sera traité pour l’épilepsie avec un médicament antiépileptique (phénobarbital ou carbamazépine ou sodium valproate). Une fois les analyses effectuées, vous recevrez une dose d’ivermectine selon votre poids, soit directement ou après 4 mois.

Après les tests de début d’étude indiqué ci-dessus, il vous sera demandé de revenir pour une visite médicale à 1, 2, 3, 4, 8 et 12 mois. A tout moment, vous devrez également informer l’équipe de recherche si vous vous sentez malade.

**Risques et inconvénients**

Les risques associés à cette étude sont rares. Le prélèvement de sang pourrait causer un inconfort et des ecchymoses de façon occasionnelle au point de piqûre. Rarement, un évanouissement ou une infection peuvent être la conséquence d’un prélèvement sanguin. Nous désinfecterons votre doigt ou votre bras avec de l’alcool avant de procéder au prélèvement et cela avec des seringues stériles et neuves chaque fois. Les prélèvements de la peau peuvent également causer un peu de douleur mais ne représentent aucun danger particulier. Le prélèvement de morceaux de peau peut également causer un peu de douleur au moment du prélèvement. Nous désinfecterons votre peau avec de l’alcool avant la procédure et utiliserons des pinces stérilisées.

Nous prévoyons que certains parmi ceux qui seront traités par l’ivermectine développeront des effets indésirables mineurs qui peuvent être des maux de tête, des douleurs abdominales, des douleurs articulaires et musculaires, des démangeaisons, des œdèmes du visage, des pieds, des bras, une faiblesse, de la fièvre, une tachycardie ou des difficultés respiratoires ou des malaises etc. Nous ne prévoyons aucune réaction adverse grave et nous prévoyons que tous ces événements disparaitront en quelques jours

Bien qu’il n’y ait pas d’évidence que l’ivermectine soit nocive pendant la grossesse et l’allaitement, par mesure de précaution, elle n’est pas recommandée pendant la grossesse et chez la femme allaitante parce qu’il n’existe pas de données fiables. Par conséquent, la femme enceinte et celle allaitante seront retirées de cette étude. Les personnes avec infection par les vers de *Loa loa* peuvent développer une encéphalopathie (maladie du cerveau) et, de ce fait,

il est prévu de retirer ces personnes avec *Loa loa* de cette étude.

Les données collectées seront traitées de façon confidentielle, c’est-à-dire qu’elles ne seront accessibles qu’à vous, aux personnes qui vous soignent et aux chercheurs de l’étude, qui sont tous soumis au secret médical. Aucune information ne sera communiquée à vos proches; c’est vous uniquement qui décidez qui vous souhaitez informer de votre santé. Vous avez également le droit à demander de voir les données qu’on a obtenu de vous et de demander éventuellement de les modifier ou de les supprimer.

**Avantages**

On vous donnera un traitement pour l’épilepsie. Les résultats des examens de sang, et de la peau qui sont utiles pour un traitement éventuel vous seront communiqués. Si l’examen de la peau montre la présence de vers un traitement par ivermectine sera donné.

**Alternatives à la participation à l’étude**

Si vous ne souhaitez pas participer à l’étude, vous pourrez toutefois recevoir le traitement à l’ivermectine (si vous avez l’onchocercose) et un traitement pour l’épilepsie. Vous pouvez aussi avoir accès aux médecins de l’étude qui seront dans le village pendant la durée de l’étude.

**Conflit d’intérêt**

Les investigateurs impliqués dans cette étude seront évalués chaque année pour s’assurer qu’ils n’ont pas de conflit d’intérêt.

**Echantillons stockés et recherche future**

Pendant votre participation à cette étude, les échantillons de sang seront collectés en conformité avec les techniques standards de prélèvement sanguin au niveau d’une veine de votre bras. Les échantillons seront utilisés pour les examens de l’étude. Le reste des échantillons sera gardé dans un endroit sécurisé et envoyé dans d’autres laboratoires plus perfectionnés pour d’autres tests. Les échantillons stockés seront gardés et utilisés lors d’études futures pour apprendre plus sur le traitement des infections parasitaires et les conditions médicales qui y sont associées.

En acceptant de participer à cette étude, vous ne perdez aucun droit en relation avec l’accès et la divulgation de vos données. Pour plus d’information sur ces droits, veuillez contacter le Dr. Michel Mandro au Ministère de la Santé Publique de la RDC au +243976500765 ou au +243824065190.

Etiquetage des échantillons stockés

Nous étiquèterons vos échantillons stockés avec un code que seule l’équipe de recherche pourra faire le lien avec à vous. Nous garderons confidentiel toute information qui peut permettre de remonter jusqu'à vous dans les conditions régies par la loi.

Etude futures

D’autres investigateurs pourraient vouloir étudier vos échantillons stockés ou vos données collectées. Dans ce cas, l’équipe du Ministère de la Santé Publique de la RDC et de l’Université d’Anvers pourraient leurs envoyer vos échantillons sans aucune information qui pourrait permettre de vous identifier. L’équipe de recherche pourrait aussi partager des informations comme le sexe, l’âge, vos antécédents de santé. Dans quelques cas, un comité d’éthique approuvera un nouveau protocole qui veut utiliser vos échantillons. Le comité d’éthique est un comité qui évalue les études médicales afin de protéger le bien-être et les droits des volontaires.

Les investigateurs utiliseront vos échantillons seulement pour la recherche. D’autres études futures pourraient avoir besoin d’informations sur votre santé que nous n’avons pas collectées pour l’étude. Dans cette situation, le Ministère de la Santé Publique de la RDC et de l’Université d’Anvers vous contacteront pour recueillir ces informations.

**Comité d’éthique**

Cette enquête a été approuvée par le Comité d’Éthique de l’Ecole de Santé de l’Université de Kinshasa et de l’Université d’Anvers, Anvers, Belgique. Ces Comités d’Éthique sont composés d’un groupe de personnes extérieures à l’enquête, des experts dans les domaines concernés par l’étude, et qui s’assurent qu’elle sera menée de la manière la plus sûre et éthique possible en respectant votre intégrité physique et morale, et vos données personnelles.

**Compensation**

Vous n’aurez pas à payer pour participer à cette étude, et vous serez motivé pour le temps pris pour participer aux activités de cette étude. La valeur de cette compensation sera d’environs de sucre ou de céréales pour une journée de travail perdue ($ US 5 ). La quantité et le type exacte de nourriture que vous recevrez seront décidés à la réunion de village tenue avant le début de l’étude.

**Rapport sur les résultats de l’étude et la confidentialité**

Les résultats de cette étude pourraient être partagés lors de réunions scientifiques ou dans des revues médicales mais votre nom ne sera pas divulgué dans le rapport. En plus, votre dossier médical et les informations médicales vous concernant seront gardés confidentiels. Toutes les utilisations imprévues de vos informations médicales feront l’objet d’un rapport aux comités d’éthique du Ministère de la Santé Publique de la RDC et de l’université d’Anvers en Belgique.

**Faire votre choix**

Si vous acceptez de participer à cette étude, vous donnez alors la permission de collecter des informations et prendre des échantillons sur vous pour de futures recherches. Quelle que soit votre décision, vous pourrez prendre part à d’autres études conduites par le Ministère de la Santé Publique de la RDC/l’Université d’Anvers. Cependant votre refus de nous laisser collecter des informations et des échantillons sur vous pourrait conduire à vous exclure de cette étude spécifique. Même si vous acceptez maintenant que nous collections des informations et des échantillons sur vous, vous avez le droit de changer d’avis plus tard. Si tel est le cas, veuillez nous contacter et dites que vous ne souhaitez plus que nous utilisons vos échantillons dans des recherches futures.

**Avez-vous des questions au sujet de votre participation à cette étude?**

Si vous avez des questions ou des soucis plus tard, vous pourrez discuter avec un des membres de notre équipe ou vous pouvez appeler le Dr Michel Mandro: 0976500765 ou 0824065190

Si vous acceptez de participer à cette étude, veuillez signer ou apposer votre empreinte digitale au bas de cette page.

FORMULAIRE DE CONSENTEMENT ÉCLAIRÉ POUR LE PARTICIPANT

**Traitement à l’ivermectine de patients avec Epilepsie associée à l’Onchocercose: Un essai clinique randomisé et contrôlé (EAOTRAIT extra)** FORMULAIRE DE CONSENTEMENT ÉCLAIRÉ POUR LE PARTICIPANT (âgé de 18 ans)

Nom du volontaire: ______________________

Numéro d’identification: __ __ __ -__ __ __ __ Date: __ __ /__ __ __ /__ __ __

| ***Partie exclusivement réservée au participant***  Je soussigné(e) certifie avoir été informé(e) concernant l’étude et avoir reçu une copie de la note d’information, ainsi que du formulaire de Consentement éclairé. J’ai compris les informations que ces documents contiennent. J’ai reçu suffisamment d’informations concernant les objectifs, le déroulement et les risques de l’étude. En outre, j’ai pu disposer d’un temps suffisant pour réfléchir sur les informations qui m’ont été communiquées et pour poser des questions, auxquelles j’ai reçu des réponses satisfaisantes. Je décide de prendre part de façon volontaire à cette étude clinique. Je peux, à tout moment et sans avoir à fournir de justification, révoquer mon Consentement, sans en subir d’inconvénient pour le suivi médical ultérieur.  Je consens librement à participer à cette étude et je consens à effectuer l’ensemble des examens requis (y compris prise de sang, échantillon d’urine et prélèvement de 2 morceaux de peau). Je suis prêt(e) à fournir toutes les informations médicales nécessaires. Je suis d’accord que des données soient conservées pour la recherche, et que des échantillons soient conservés et envoyés dans des laboratoires de référence Congolais ou étrangers pour rechercher la cause de l’épilepsie ou des facteurs qui peuvent favoriser le développement de l’épilepsie.  🞏 Oui 🞏 Non  J’accepte que mon médecin habituel soit informé de ma participation à cette étude. Je comprends que si j’ai des questions sur l’étude ou si je ne veux plus y participer, je peux contacter à tout moment le chercheur responsable.  **Date : …………………………… Nom : .......……………….........................……………**  **Signature (ou empreinte digitale. du participant : …………………………………………...**  **Date : ……………………………Nom du témoin : ……………………………………..**  **Signature du témoin : ……………………………………** |
| --- |

| ***Partie destinée exclusivement à l’équipe de l’investigateur***  Je, soussigné (e), ______________________________________confirme avoir informé le participant de tous les aspects pertinents de la présente étude. Je confirme qu’il/elle a consenti volontairement à participer à l’étude.  Le médecin/investigateur local de l’étude à_________________________________________________________  **Date : ____________________________________ Signature :** |
| --- |

**Traitement à l’ivermectine de patients avec Epilepsie associée à l’Onchocercose: Un essai clinique randomisé et contrôlé (EAOTRAIT extra)** FORMULAIRE DE CONSENTEMENT ÉCLAIRÉ POUR LE PARENT OU RESPONSABLE DU PARTICIPANT

Nom du volontaire (enfant): ______________________

Nom du parent ou responsable pour l’enfant_______________________________________

Relation avec l’enfant : _______________________________________________________

Numéro d’identification: __ __ __ -__ __ __ __ Date: __ __ /__ __ __ /__ __ __

| ***Partie exclusivement réservée au parent ou responsable du participant***  Je soussigné(e) certifie avoir été informé(e) concernant l’étude et avoir reçu une copie de la note d’information, ainsi que du formulaire de Consentement éclairé. J’ai compris les informations que ces documents contiennent. J’ai reçu suffisamment d’informations concernant les objectifs, le déroulement et les risques de l’étude. En outre, j’ai pu disposer d’un temps suffisant pour réfléchir sur les informations qui m’ont été communiquées et pour poser des questions, auxquelles j’ai reçu des réponses satisfaisantes. Je décide que mon enfant peut prendre part de façon volontaire à cette étude clinique. Je peux, à tout moment et sans avoir à fournir de justification, révoquer mon Consentement, sans en subir d’inconvénient pour le suivi médical ultérieur.  Je consens librement que mon enfant participe à cette étude et je consens à effectuer l’ensemble des examens requis (y compris prise de sang, échantillon d’urine et prélèvement de 2 morceaux de peau). Je suis prêt(e) à fournir toutes les informations médicales nécessaires. Je suis d’accord que des données soient conservées pour la recherche, et que des échantillons soient conservés et envoyés dans des laboratoires de référence Congolais ou étrangers pour rechercher la cause de l’épilepsie ou des facteurs qui peuvent favoriser le développement de l’épilepsie.  🞏 Oui 🞏 Non  J’accepte que le médecin habituel de l’enfant soit informé de sa participation à cette étude. Je comprends que si j’ai des questions sur l’étude ou si je ne veux plus y participer, je peux contacter à tout moment le chercheur responsable.  **Date : …………………………… Nom : .......……………….........................……………**  **Signature (ou empreinte digitale. du participant) : …………………………………………...**  **Date : ……………………………Nom du témoin : ……………………………………..**  **Signature du témoin : ……………………………………** |
| --- |

| ***Partie destinée exclusivement à l’équipe de l’investigateur***  Je, soussigné (e), ______________________________________confirme avoir informé le participant, le parent ou le responsable du participant de tous les aspects pertinents de la présente étude. Je confirme qu’il/elle a consenti volontairement à participer à l’étude.  Le médecin/investigateur local de l’étude à_________________________________________________________  **Date : ____________________________________ Signature :** |
| --- |

**Traitement à l’ivermectine de patients avec Epilepsie associée à l’Onchocercose: Un essai clinique randomisé et contrôlé (EAOTRAIT** **extra)** FORMULAIRE DE ASSENTIMENT ÉCLAIRÉ POUR LE PARTICIPANT (enfant de 12 à 18 ans)

Nom du volontaire (enfant): ______________________

Numéro d’identification: __ __ __ -__ __ __ __ Date: __ __ /__ __ __ /__ __ __

| ***Partie exclusivement réservée au participant***  Je soussigné(e) certifie avoir été informé(e) concernant l’étude et avoir reçu une copie de la note d’information, ainsi que du formulaire de assentiment éclairé. J’ai compris les informations que ces documents contiennent. J’ai reçu suffisamment d’informations concernant les objectifs, le déroulement et les risques de l’étude. En outre, j’ai pu disposer d’un temps suffisant pour réfléchir sur les informations qui m’ont été communiquées et pour poser des questions, auxquelles j’ai reçu des réponses satisfaisantes. Je décide que je veux prendre part de façon volontaire à cette étude clinique. Je peux, à tout moment et sans avoir à fournir de justification, révoquer mon Consentement, sans en subir d’inconvénient pour le suivi médical ultérieur.  Je consens librement de participer à cette étude et je consens à effectuer l’ensemble des examens requis (y compris prise de sang, échantillon d’urine et prélèvement de 2 morceaux de peau). Je suis prêt(e) à fournir toutes les informations médicales nécessaires. Je suis d’accord que des données soient conservées pour la recherche, et que des échantillons soient conservés et envoyés dans des laboratoires de référence Congolais ou étrangers pour rechercher la cause de l’épilepsie ou des facteurs qui peuvent favoriser le développement de l’épilepsie.  🞏 Oui 🞏 Non  J’accepte que mon médecin habituel soit informé de ma participation à cette étude. Je comprends que si j’ai des questions sur l’étude ou si je ne veux plus y participer, je peux contacter à tout moment le chercheur responsable.  **Date : …………………………… Nom : .......……………….........................……………**  **Signature (ou empreinte digitale. du participant : …………………………………………...**  **Date : ……………………………Nom du témoin : ……………………………………..**  **Signature du témoin : ……………………………………** |
| --- |

| ***Partie destinée exclusivement à l’équipe de l’investigateur***  Je, soussigné (e), ______________________________________confirme avoir informé le participant de tous les aspects pertinents de la présente étude. Je confirme qu’il/elle a consenti volontairement à participer à l’étude.  Le médecin/investigateur local de l’étude à_________________________________________________________  **Date : ____________________________________ Signature :** |
| --- |
