# Supplementary material for: Single versus Multiple Dose Ivermectin Regimen in Onchocerciasis-Infected Persons with Epilepsy Treated with Phenobarbital: A Randomized Clinical Trial in the Democratic Republic of Congo
Source: Pathogens. 2020 Mar 10;9(3):205. doi: 10.3390/pathogens9030205 (PMC7157533; doi:10.3390/pathogens9030205)
Supplement: Supplementary file 1 [file pathogens-09-00205-s001.zip › S3 ZIP model.docx]

**Supplementary Material S3**

**Data analysis using a Zero-inflated Poisson regression model**

Adjusting for other covariates, logistic regression from Zero-inflated Poisson regression revealed that intake of ivermectin twice and thrice per year resulted in higher odds of seizure freedom during the last four months of the trial as compared to an annual ivermectin regimen. In Poisson model component the rate of the seizure counts during the last four months was significantly lower in PWE treated twice and thrice compared to those treated once.

**Table S3**. Zero-inflated Poisson regression exploring the association between ivermectin once, twice and thrice on number of seizure during the last four months adjusted for study group (group 1 and 2 combined)

|  | **Zero-Inflated Odds of Seizure freedom during last 4 months** | | | | **Seizure frequency**  **IRR Poisson Model** | | | |
| --- | --- | --- | --- | --- | --- | --- | --- | --- |
| **Variables** | **OR** | **95% CI** | | **P-value** | **IRR** | **95% CI** | | **P-value** |
| Ivermectin dose twice/year vs once/year | 1.872 | 1.209 | 2.899 | 0.005 | 0.134 | 0.026 | 0.682 | 0.015 |
| Ivermectin dose thrice/year vs once/year | 1.159 | 0.917 | 1.466 | 0.217 | 0.297 | 0.095 | 0.923 | 0.036 |
| Female vs male | 0.615 | 0.506 | 0.747 | <0.001 | 0.576 | 0.257 | 1.289 | 0.180 |
| Age | 0.987 | 0.967 | 1.007 | 0.199 | 0.994 | 1.047 | 0.944 | 0.828 |
| Weight (in kg) | 1.009 | 0.998 | 1.020 | 0.128 | 1.003 | 1.048 | 0.959 | 0.897 |
| Seizure frequency at baseline | 1.647 | 1.444 | 1.878 | <0.001 | 1.120 | 1.942 | 0.646 | 0.687 |
| Duration of epilepsy (years) | 0.764 | 0.674 | 0.865 | <0.001 | 1.028 | 1.718 | 0.616 | 0.915 |
| Microfilarial density at baseline | 1.100 | 1.040 | 1.164 | 0.001 | 1.316 | 1.640 | 1.056 | 0.014 |
| Optimal vs sub-optimal AED adherence during last four months | 0.850 | 0.688 | 1.049 | 0.131 | 0.410 | 0.171 | 0.981 | 0.045 |
| >30mg of AED increase (between M0 and M12) vs no increase | 1.044 | 0.837 | 1.303 | 0.699 | 7.584 | 2.871 | 20.033 | <0.001 |
| Study group 1 vs group 2 | 0.729 | 0.537 | 0.991 | 0.044 | 0.322 | 0.094 | 1.106 | 0.072 |
| ***CI****: confidence interval;* ***OR****: Odd’s Ratios;* ***IRR****:  incidence rate ratio.* | | | | | | | | |
